# A New Growing Slide

**Published:** 1869-06

**Authors:** 


A NEW GROWING SLIDE, which is simple and convenient, is described by Mr. J. C. Muller, in the *Monthly Microscopical Journal* of March. Any ordinary glass-slide is pierced with a minute hole, at about three-tenths of an inch from the centre on one side. When an object under investigation is put upon it immersed in water, the thin glass cover is so placed as to include this hole, which may be near the margin of the disc. When it is desired to keep the specimen moist while off the stage of the microscope, the slide is placed in the undermentioned piece of apparatus; viz., a flat trough 7 inches long, 2½ inches wide, with straight sides, ¾ of an inch high. In this slide is placed uppermost, with one end (that nearest the hole) resting against the bottom of the vessel on one side, and the other end resting upon the edge of it. Sufficient water is put into the vessel to admit of the liquid reaching within a quarter or half an inch of the glass cover on the uppermost side, when it will be found, that by capillary attraction, the water on the underside reaches beyond the center of the slide, and consequently beyond the hole with which it is pierced. In this state the object will remain moist as long as the trough contains a sufficient quantity of water. When required to be placed on the stage of the microscope, the water is easily wiped off without disturbing the object.

